# Closing the gaps between science, recommendations and practice of physical activity in rheumatoid arthritis

**DOI:** 10.1093/rap/rkaf147

**Published:** 2025-12-23

**Authors:** William J Gregory, Carol McCrum

**Affiliations:** Rheumatology Department, Salford Royal Hospital, Northern Care Alliance NHS Foundation Trust, Salford, UK; Faculty of Health and Education, Manchester Metropolitan University, Manchester, UK; University of Brighton, Centre for Health Professions Research, Eastbourne, UK; Rheumatology Department, Canberra Health Service, Canberra, ACT, Australia

**This editorial refers to ‘Understanding physical activity participation in people living with rheumatoid arthritis’ by Julia New-Tolley *et al.*, 2026; https://doi.org/10.1093/rap/rkaf146.** 

In the New-Tolley *et al.* [1] article in this issue of *Rheumatology Advances in Practice* there is an exploration of both participation and views on physical activity of people living with RA. A comparison is made with World Health Organization (WHO) guidelines on physical activity and sedentary behaviour for this Australian cohort of people living with RA. Using an online questionnaire, the study assessed the type, frequency and intensity of physical activity, as well as perceptions of exercises and its barriers and enablers.

An important insight of this research to consider is that while 67% of participants reported achieving the recommended levels of aerobic activity, only 24% undertook the recommended level of both aerobic exercise and muscle strengthening. Participants who reported meeting physical activity guidelines were more likely to have lower disease activity, less fatigue and greater confidence in exercising safely. They were also more likely to have received ‘useful advice’ from healthcare professionals.

The study has highlighted a significant gap in muscle-strengthening activity, with many participants unaware of how to incorporate muscle-strengthening exercises or doubting its feasibility with their RA. This research underscores the need for targeted interventions to promote physical activity, with particular emphasis on muscle-strengthening activities in supporting those living with RA. Findings suggests that rheumatology teams should play a more active role in counselling on undertaking physical activity and strengthening, particularly in addressing safety concerns, building confidence and supporting incorporation into a daily routine. The findings support the integration of allied health professionals and tailored education into routine RA management to bridge the gap between physical activity recommendations and real-world practice. There is a need to enable access to appropriate exercise facilities for those living with RA as well as considering what physical activities could be undertaken at home or as a part of usual daily routines. The UK National Institute of Health and Care Excellence Guideline on RA surveillance review [2] in 2023 recommended incorporation of aerobic exercise and resistance training but did not specifically state frequency or intensity; meanwhile, the WHO Fact Sheet on RA [3] merely states that optimal functioning should be aimed for via rehabilitation input.

Research has shown that physical activity is not just safe but also beneficial for people living with RA. Metsios *et al.* [4] emphasize that aerobic and resistance training improve important functional outcomes, potentially by blunting the inflammatory response. In addition, New-Tolley *et al.* [1] report that some of their RA participants rated regular physical activity as a mood enhancer, however, most participants did not think it possible to engage in regular muscle-strengthening activities or exercise of moderate to vigorous intensity.

These findings position physical activity and exercise as recommended but often underutilized treatments that complement pharmacological treatments by directly addressing inflammation and disability. The physiological mechanisms underpinning these improvements are linked to changes in cytokine activity; exercise has been shown to modulate levels of TNFs and interleukins, reducing systemic inflammation [4]. Stavropoulos-Kalinoglou *et al.* [5] showed that reductions in adiposity following a 6-month individualized program was associated with decreases in C-reactive protein, a key marker of inflammation. These findings highlight how exercise influences immune pathways, improving immunosurveillance and overall immunocompetence.

Beyond molecular changes, consistent positive associations have been found between regular physical activity and quality of life and functional capabilities in RA populations [6, 7]. Long-standing, supervised exercise therapy has been shown to be more effective than usual care, particularly for individuals with severe functional limitations [8]. Aerobic, resistance and neuromotor exercises—when tailored to disease activity and symptoms—enhance strength, balance and daily function, supporting patients in managing fatigue, pain and mobility challenges [6]. In addition, physiotherapy input in RA is a cost-effective intervention [8, 9].

From the patient perspective, exercise and regular physical activity contribute to pain reduction, improved joint function, weight management, better sleep and enhanced mood. New Tolley *et al.* [1] add evidence on the importance of stressing that exercise programs must be individualised, sustainable and enjoyable to ensure adherence. Patients are encouraged to consider accountability, social support and strategies for flare-ups, aligning their routines with both general and RA-specific guidelines to maximize long-term benefit.

Accumulating research in populations of people living with RA and in our understanding of exercise science in relation to long-term inflammatory conditions is highlighting how important it is to enable and empower people with RA to be active and to have confidence to undertake both aerobic and muscle strengthening activities regularly. A UK survey of rheumatology physiotherapists in 2022 showed that ‘exercise prescription’ was the most common task undertaken by this professional group [10]. There are other professional groups who engage with people living with RA and have a clear role in encouraging and improving physical activity levels. It has recently been suggested that the initial advice about physical activity given at the diagnosis of RA has a significant impact on future exercise behaviours [11]. All healthcare professionals have a role in advocating for lifelong physical activity engagement for those living with RA. Resources such as Moving Medicine [12] encourage and enable all health professionals to utilise even very short periods in their consultations with RA patients to check, reassure and advocate for physical activity habits.

There is a clear need to upskill and support all health professionals involved in the care of people living with RA to become better advocates for the role of regular physical activity and to consider how they might assess and engage their patients in developing habits of regular physical activity.

## Data Availability

No new data were generated or analysed in support of this editorial.
